# Air trapping in COVID-19 patients following hospital discharge: retrospective evaluation with paired inspiratory/expiratory thin-section CT

**DOI:** 10.1007/s00330-022-08580-2

**Published:** 2022-02-28

**Authors:** Tomás Franquet, Ana Giménez, Loren Ketai, Sandra Mazzini, Andrea Rial, Virginia Pomar, Pere Domingo

**Affiliations:** 1 Department of Radiology, Hospital de la Santa Creu i Sant Pau, Barcelona, Spain; 2 Department of Medicine, Universitat Autónoma de Barcelona, Barcelona, Spain; 3 Department of Radiology, University of New Mexico Health Science Center, Albuquerque, NM USA; 4 Department of Pneumology, Hospital de la Santa Creu i Sant Pau, Barcelona, Spain; 5 Department of Infectious Diseases, Hospital de la Santa Creu i Sant Pau, Barcelona, Spain; 6 Institut de Recerca Biomèdica del Hospital de Sant Pau, Barcelona, Spain

**Keywords:** Air-trapping, Long-COVID, Inspiratory

## Abstract

**Objectives:**

The study reports our experience with paired inspiration/expiration thin-section computed tomographic (CT) scans in the follow-up of COVID-19 patients with persistent respiratory symptoms.

**Methods:**

From August 13, 2020, to May 31, 2021, 48 long-COVID patients with respiratory symptoms (27 men and 21 women; median age, 62.0 years; interquartile range: 54.0–69.0 years) underwent follow-up paired inspiration-expiration thin-section CT scans. Patient demographics, length of hospital stay, intensive care unit admission rate, and clinical and laboratory features of acute infection were also included. The scans were obtained on a median of 72.5 days after onset of symptoms (interquartile range: 58.5–86.5) and at least 30 days after hospital discharge. Thin-section CT findings included ground-glass opacity, mosaic attenuation pattern, consolidation, traction bronchiectasis, reticulation, parenchymal bands, bronchial wall thickening, and air trapping. We used a quantitative score to determine the degree of air trapping in the expiratory scans.

**Results:**

Parenchymal abnormality was found in 50% (24/48) of patients and included air trapping (37/48, 77%), ground-glass opacities (19/48, 40%), reticulation (18/48, 38%), parenchymal bands (15/48, 31%), traction bronchiectasis (9/48, 19%), mosaic attenuation pattern (9/48, 19%), bronchial wall thickening (6/48, 13%), and consolidation (2/48, 4%). The absence of air trapping was observed in 11/48 (23%), mild air trapping in 20/48 (42%), moderate in 13/48 (27%), and severe in 4/48 (8%). Independent predictors of air trapping were, in decreasing order of importance, gender (*p* = 0.0085), and age (*p* = 0.0182).

**Conclusions:**

Our results, in a limited number of patients, suggest that follow-up with paired inspiratory/expiratory CT in long-COVID patients with persistent respiratory symptoms commonly displays air trapping.

**Key Points:**

• *Our experience indicates that paired inspiratory/expiratory CT in long-COVID patients with persistent respiratory symptoms commonly displays air trapping.*

• *Iterative reconstruction and dose-reduction options are recommended for demonstrating air trapping in long-COVID patients.*

## Introduction

The coronavirus disease 2019 (COVID-19) pandemic caused by the severe acute respiratory syndrome coronavirus 2 (SARS-CoV-2) arose in late December 2019 in Wuhan, Hubei Province, China [1–3]. SARS-CoV-2 has resulted in a pandemic with more than 180 million confirmed cases worldwide and a death toll of more than 3.9 million (Johns Hopkins Coronavirus Resource Center. https://coronavirus.jhu.edu/map.html).

Clinically, COVID-19 infection can range from asymptomatic or mild illness (e.g., fever with or without cough) to severe respiratory distress, multiorgan failure, and death.

The term “long-COVID” describes those patients with COVID-19 who experience persistent symptoms regardless of initial disease severity or age. Respiratory symptoms include dyspnea, shortness of breath, and repetitive cough, and it might take weeks or months to eventually recovery [2–5].

Typical and atypical imaging findings of COVID-19 infection have been extensively reported [6–8]. A meta-analysis including twenty-eight studies and 3,466 patients has been recently published to optimize the diagnostic interpretation of chest CT scanning for COVID-19 [9]. In patients without severe respiratory disease, the major pulmonary CT findings of COVID-19 are ground-glass opacities (GGOs) with a typical bilateral and multilobar distribution and middle-lower lung predominance, mainly with a subpleural distribution [10, 11]. In more severely affected patients, consolidation replaces GGO, and the abnormalities extend to the upper lobes or become bilateral or both [11].

However, little is known about sequential CT findings during the subsequent course of COVID-19, especially about sequelae that may occur during convalescence.

Expiratory CT has established itself as an essential adjunct to conventional CT, in the demonstration of air trapping in patients with suspected obstructive small airway disease [12]. In our institution, paired inspiratory/expiratory CT examination is obtained routinely to evaluate patients in whom airway disease is suspected.

The purpose of our study was to evaluate the usefulness of paired inspiratory/expiratory CT in the detection of air trapping in the follow-up of long-COVID patients presenting with respiratory complaints.

## Materials and methods

### Patient population

Between August 13, 2020, and May 31, 2021, 1722 proved COVID-19 patients were admitted to the Hospital de la Santa Creu i Sant Pau, Barcelona, Spain; 105 died, whereas 1617 were discharged. Among them, 162 patients fulfilled the clinical criteria for diagnosis of long COVID. After excluding 102 patients because of smoking or prior respiratory disease, and 12 who declined or were unable to participate, 48 previously healthy patients with respiratory symptoms were enrolled in the present study.

These patients were recruited during regularly scheduled follow-up visits. All the patients had persistent cough and/or dyspnea.

All 48 patients underwent paired inspiratory/expiratory CT after clinical recovery and were released from quarantine, between 22 and 96 days (mean, 51.8 days ± 20.2), after the onset of symptoms. In 9 out of the 48 patients, a previous conventional inspiratory CT in the acute phase of the infection was available.

Patients were divided into two groups: those with severe respiratory disease (i.e., requiring ICU admission because of ARDS) and those with mild to moderate disease (not admitted to the ICU). Adult respiratory distress syndrome (ARDS), defined according to the American-European consensus conference [10], occurred in 14 (29.2%) of 48 patients.

Demographic, clinical, and laboratory data related to the severity of the respiratory disease were recorded. Demographics include age, sex, and comorbidities (arterial hypertension, diabetes mellitus, dyslipidemia, epilepsia, and gout), clinical and radiologic data, and treatment modalities, such as corticosteroids, tocilizumab, or other immunomodulating agents. Laboratory data included peak serum C-reactive protein (CPR) levels, lactate dehydrogenase (LDH), aspartate aminotransferase (AST), d-dimer, ferritin, interleukin-6 (IL-6), Krebs von den Lungen glycoprotein (KL-6), and leukocyte differential counts.

This study was conducted with institutional review board approval and was performed during the period of convalescence, approximately 3 months after the patient’s clinical recovery from COVID-19. Written informed consent was obtained from all patients before inclusion. The study was approved by the Ethics Committee of the Hospital de la Santa Creu I Sant Pau.

### Laboratory methods

The diagnosis of SARS-CoV-2 infection was microbiologically proved in all the patients.

Nasopharyngeal swabs were obtained according to a standardized hospital protocol. Detection of SARS-CoV-2 was done through RT-PCR (Xpert® Xpress SARS-CoV-2).

### CT protocol

Before CT scanning, patients were coached in the breathing technique by a radiology technologist to hold breaths at full inspiration and full expiration. Other than verbal coaching, we did not attempt to control the patient’s respiratory status during scanning. After carefully instructing breathing, the patients were scanned at the suspended end-inspiratory and end-expiratory volume from the lung apices through the lung bases. No IV contrast medium was injected.

Images at full inspiration and end-expiration without a spirometer-controlled imaging technique were acquired using a multi-detector 16-slice scanner (Philips Brilliance CT 16 slice) with the following parameters: X-ray voltage, 120 kVp; tube current, auto exposure control (100–200 mAs); collimation, 1 mm; high-speed mode; and pitch equivalent, 1.5. The scanning field of view ranged from 28 to 44 cm, based on the subject’s body habitus. Exposure time was 0.53 s, and the matrix size was 768 × 768 pixels. Images were reconstructed with a 1.25-mm slice thickness (with 0.625 mm overlapping), using the “Bone” algorithm. For this sequence, patients were instructed to take a deep breath and hold it. After end-inspiratory scanning, the patients were coached with instructions for the dynamic expiratory component of the study. For this sequence, patients were instructed to take a deep breath and blow it out during the image acquisition, which was coordinated with, to begin with, the patient’s forced expiratory effort. A reduced-dose technique was employed to minimize radiation exposure for dynamic expiratory sequences.

### Image review and scoring

The CT images in the 48 patients in the study cohort were reviewed, and findings were scored by three chest radiologists (T.F., A.G. and S.M., who had 20, 16, and 5 years of experience in thoracic radiology, respectively) in consensus. Because the three observers did not independently review each case, interobserver agreement was not evaluated.

The major CT findings were described based on the recommendations of the Nomenclature Committee of the Fleischner Society [13]. Thin-section CT findings, including ground-glass opacity, mosaic-attenuation pattern, consolidation, traction bronchiectasis, reticulation, parenchymal bands, bronchial wall thickening, and air trapping, were recorded. Ground-glass opacity involved an increase in lung parenchymal opacification without obscuration of the underlying vessels, and consolidation involved an increase in parenchymal opacification with obscuration of the underlying vessels. Parenchymal bands were defined as non-tapering linear opacities a few millimeters thick and several centimeters long. Air trapping was defined as an area of low attenuation in contrast with the background attenuation of lung parenchyma on images obtained during expiration.

Satisfactory CT examinations were based on a qualitative visual assessment of lung parenchyma and the decreased cross-sectional area between maximum inspiration and maximum expiration on CT.

Areas of air trapping were present when lung regions failed to increase in attenuation, as compared with findings on inspiratory scan at or near the same level [13, 14]. All paired inspiratory/expiratory CT scans were reviewed directly from the workstation by three thoracic radiologists aware that all patients had proven COVID-19 infection but were otherwise blinded to other clinical information.

All thin-section CT scans were reviewed at a window width and level of 1000–1500 HU and −500 to −650 HU, respectively, for lung parenchyma, and 300–350 HU and 20–50 HU, respectively, for soft tissue and mediastinum, by using image data that complied with the Digital Imaging and Communications in Medicine standard.

The degree of air trapping within each lung zone was evaluated by scoring the CT images using a 4-point scale based on visual assessment (Table 1). Each lung was divided into three lung zones: upper (above the carina), middle (below the carina up to the inferior pulmonary vein), and lower (below the inferior pulmonary vein) zones. Each lung zone (total of six lung zones) was assigned a score as follows: score 0 (no air trapping visible); score 1 (1–25% of the cross-sectional area of lung affected), score 2 (26–50% affected; score 3) (51–75% affected), and score 4 (76–100% affected). The total air trapping score was obtained by summing six scores. The scores were added to provide a total CT severity score cumulative score ranging from 0 to 24. Thus, for each lung, the maximum possible score was 12 (three levels times four points at each level), and for both lungs, 24.

**Table 1:** System for scoring air trapping in expiratory thin-section CT scans. Each lung was divided in three lung zones: upper, middle, and lower. The maximum score for each lung was 12 points. Both lungs had a global maximum score of 24 points

Score	Definition
0	None
1	1 – 25% of air trapping in each lung zone
2	26 – 50% of air trapping in each lung zone
3	51 – 75% of air trapping in each lung zone
4	> 75% of air trapping in each lung zone

### Statistical analyses

All the data were analyzed with statistical software (SPSS, version 11.0; SPSS). The Mann-Whitney test was used to analyze differences in ground-glass opacity, and air trapping scores between patient subgroups based on binary variables (male vs female sex, with vs. without ARDS and steroid therapy). Spearman rank correlation was performed to analyze the relationship between continuous variables (age, peak levels of CRP, LDH, and AST) and categoric variables (thin-section CT scores). All values were expressed as the median and interquartile range (IQR) unless otherwise indicated. A *p* value of less than .05 was considered to indicate a statistically significant difference for all test results. A multivariable logistic regression model was used to identify factors independently associated with air trapping. Any variable tested in univariate analysis with a *p* < 0.25 and all known clinical significance variables was selected as the first multivariate model candidates. We then followed the purposeful selection of covariates method described by Hosmer et al [15]. Final parameter estimates are shown as odds ratios (ORs) with their corresponding 95% confidence intervals (CIs). Data were analyzed using IBM® SPSS®, statistics, version 26.0.

## Results

### Demographics, clinical, and laboratory data

Forty-eight symptomatic patients were recruited during regularly scheduled follow-up visits. All the patients presented with persistent cough and/or dyspnea or both at the examination. There were 27 men and 21 women with a median age of 62 (IQR: 54–69) years (range: 31–79). Twenty-seven patients (56.2%) had comorbidities predisposing to SARS-CoV-2 infection, the most common being arterial hypertension (16, 33.3%). The median Charlson index was 0.0 (IQR: 0.0–0.0). Fourteen patients (29.2%) developed adult distress respiratory syndrome (ARDS). Demographics, clinical, and laboratory data are summarized in Table 2.

**Table 2:** Demographic, clinical, and laboratory data in patients with persistent dyspnea and cough after acute COVID-19

Characteristic	With ARDS (*N* = 14)	Without ARDS (*N* = 34)	*p* value
Age, median, year	66.0 (62.0 – 75.0)	55.5 (47.0 – 65.0)	0.0027
Male, *n* (%)	9 (64.3)	18 (52.9)	0.5361
Charlson comorbidity index	0.0 (0.0 – 0.0)	0.0 (0.0 – 0.0)	0.4631
Comorbidities, %	9 (64.3)	18 (52.9)	0.5361
Arterial hypertension, *n* (%)	6 (42.8)	10 (29.4)	0.5026
Diabetes mellitus, *n* (%)	0 (0)	3 (8.8)	0.5459
Other*, *n* (%)	3 (21.4)	3 (8.8)	0.4714
Interval onset-admission, days	7.5 (6.0 – 10.0)	7.0 (4.0 – 7.0)	0.1306
Symptoms during acute COVID-19			
Fever, *n* (%)	14 (100)	34 (100)	–
Dyspnea, *n* (%)	5 (35.7)	25 (73.5)	0.0219
Anosmia/ageusia, *n* (%)	4 (28,6)	9 (26.5)	0.8349
Cough, *n* (%)	4 (28,6)	27 (79,4)	0.0019
Fatigue, *n* (%)	2 (14.3)	12 (35.3)	0.1812
Diarrhea, *n* (%)	2 (14.3)	13 (38.2)	0.0759
Arthromyalgia, *n* (%)	3 (21.4)	5 (14.7)	0.8870
Headache, *n* (%)	2 (14.3)	7 (20.6)	0.9184
Rash, *n* (%)	0 (0)	3 (8.8)	–
Abdominal pain, *n* (%)	2 (14.3)	0 (0)	–
O_2_ saturation, %	92.0 (92.0 – 93.0)	94.5 (92.0 – 98.0)	0.0151
PaO_2_/FiO_2_ ratio	173.0 (69.0 – 385.0)	304.5 (261.0 – 348.0)	0.0656
C-reactive protein, mg/L	226.5 (201.0 – 256.0)	97.7 (63.1 – 148.0)	< 0.0001
AST, IU/l	54.0 (31.0 – 79.0)	51.5 (28.0 – 100.0)	0.5243
ALT, IU/l	66.0 (54.0 – 79.0)	53.0 (31.0 – 87.0)	0.3506
LDH, IU/l	541.0 (367.0 – 654.0)	299.0 (179.0 – 504.0)	0.0008
D dimer, ng/mL	633.0 (558.0 – 665.0)	560.0 (243.0 – 862.0)	0.2857
KL6, U/L	782.0 (138.0 – 941.7)	184.0 (144.7 – 331.0)	0.5715
IL-6, pg/mL	2094.0 (73.6 – 5573.0)	42.0 (18.0 – 116.0)	0.0103
Ferritin, ng/mL	2852.5 (1343.0 – 3771.0)	747.0 (336.0 – 1691.0)	0.0006
Neutrophil/lymphocyte ratio	7.95 (5.95 – 17.76)	2.9 (1.9 – 5.0)	< 0.0001
Therapeutics			
Dexamethasone, *n* (%)	14 (100)	26 (76.5)	–
Tocilizumab, *n* (%)	10 (71.4)	15 (44.1)	0.1603
Other**, *n* (%)	13 (92.8)	18 (52.9)	0.0216
Air trapping score	12.5 (12.0 – 14.0)	10.0 (8.0 – 12.0)	0.0028

All values expressed as median and interquartile range (IQR) unless otherwise specified. ^*^Includes dyslipidemia [4], epilepsia [1], and gout [1]

^**^Includes antibiotics [29], hemodialysis [2], and ECMO [2]

*ARDS* adult respiratory distress syndrome, *O*_*2*_ oxygen, *PaO*_*2*_ partial pressure of oxygen in arterial blood, *FiO*_*2*_ fraction of inspired oxygen, *AST* aspartate aminotransferase, *ALT* alanine aminotransferase, *LDH* lactate dehydrogenase, *KL6* Krebs von den Lungen glycoprotein, *IL-6* interleukin 6

### CT findings

**Figure 1:**
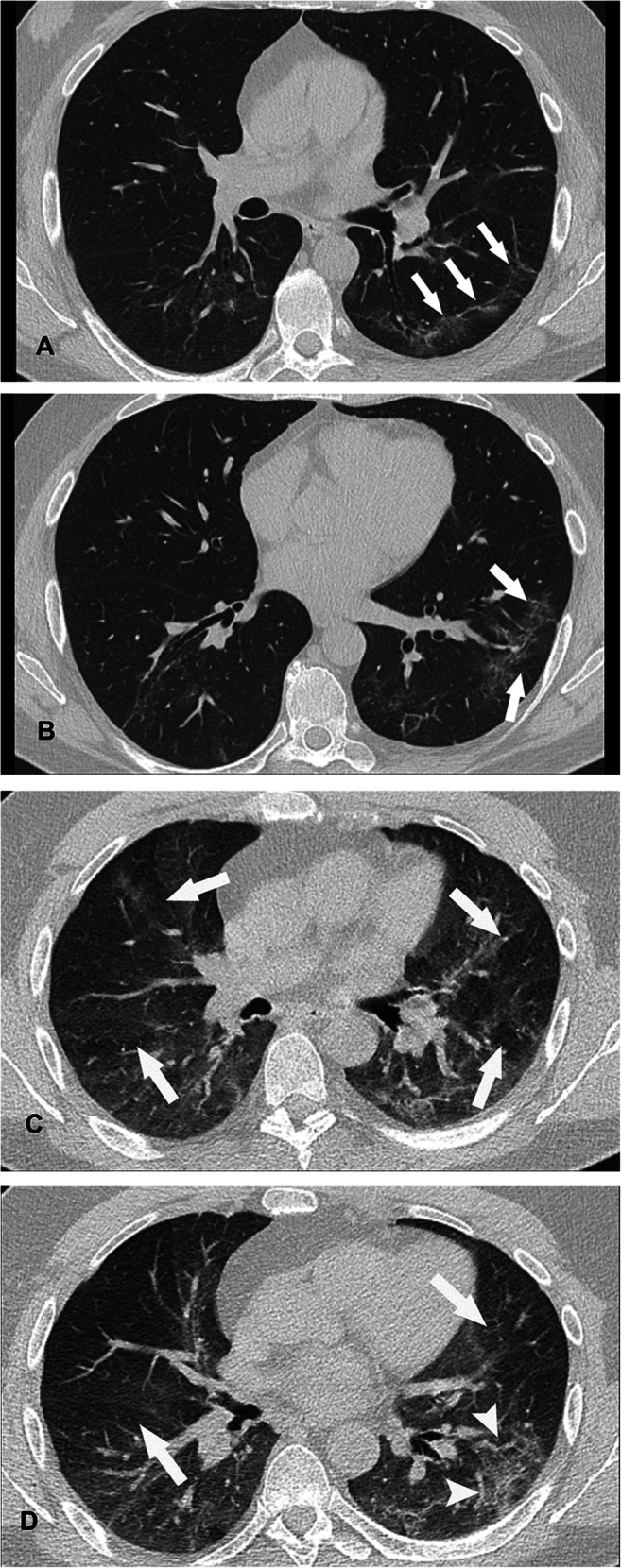
**A**, **B** Transverse CT scans in a 47-year-old nonsmoking man who had COVID-19 obtained during inspiration at follow-up examination, 70 days after dyspnea and shortness of breath, show minimal peripheral ground-glass opacities (arrows); **C**, **D** expiratory transverse CT images at the same levels show linear consolidation in the subpleural region of the left lower lobe (arrowheads) and bilateral multifocal areas of low attenuation due to air trapping (arrows) that takes up the entire segments. Note that the same levels appear normal on the inspiratory images

**Figure 2:**
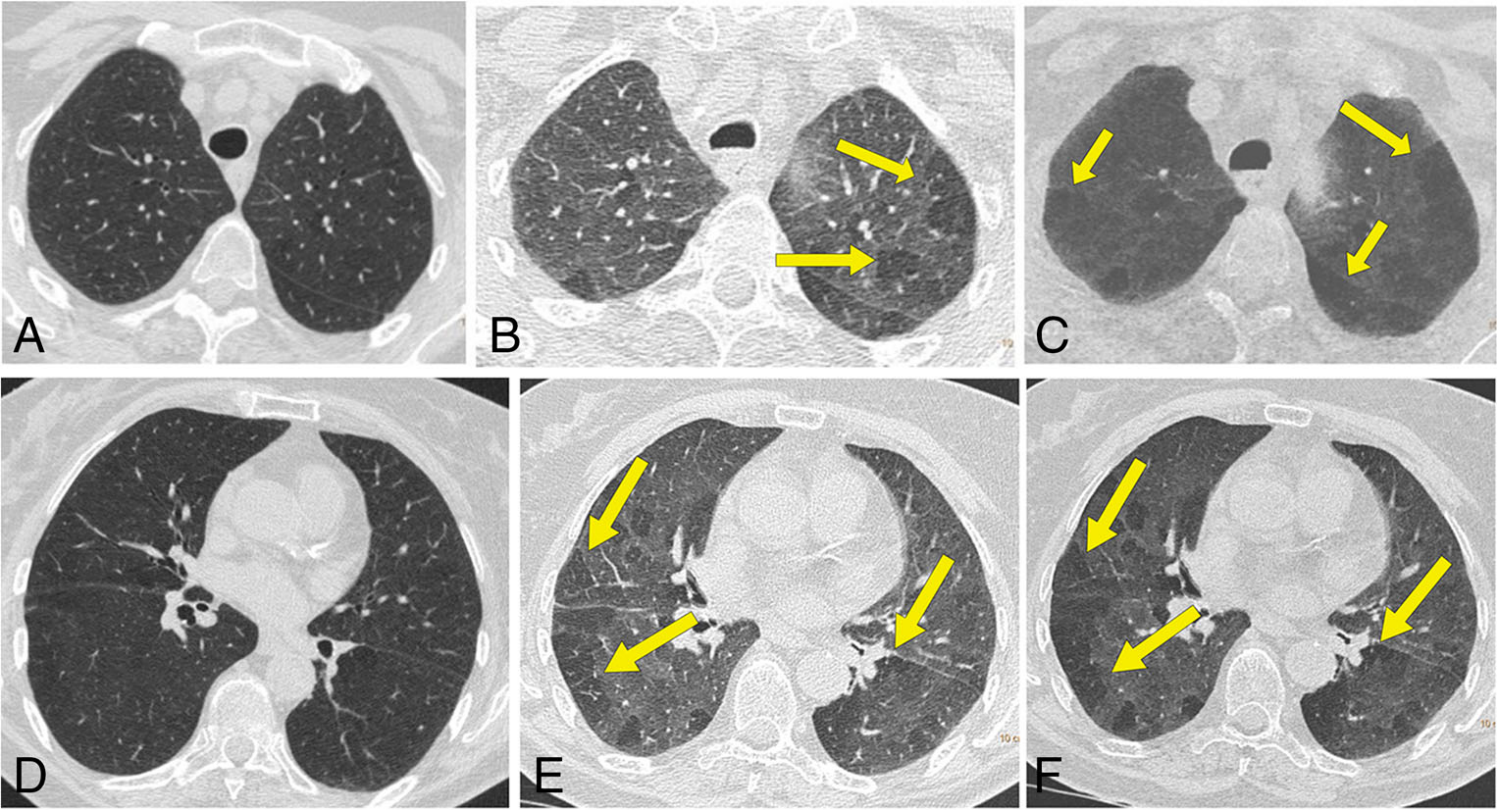
A 67-year-old nonsmoking man who had COVID-19. Inspiratory CT and expiratory CT scans at follow-up examination, 50 days after dyspnea and shortness of breath. **A** Inspiratory CT scan shows no abnormalities. **B** Expiratory CT scan obtained at same level as **A** shows significant multifocal areas of low attenuation due to air trapping in both upper lobes (arrows). **C** MiniP reformatted image is useful to improve the degree of air trapping (arrows). **D** Inspiratory CT scan at the level of the lower lobes shows no abnormality. **E** Expiratory CT scan obtained at same level as **D** shows significant multifocal areas of low attenuation due to air trapping in both lower lobes (arrows). **F** MiniP reformatted image also shows bilateral air trapping (arrows)

**Figure 3:**
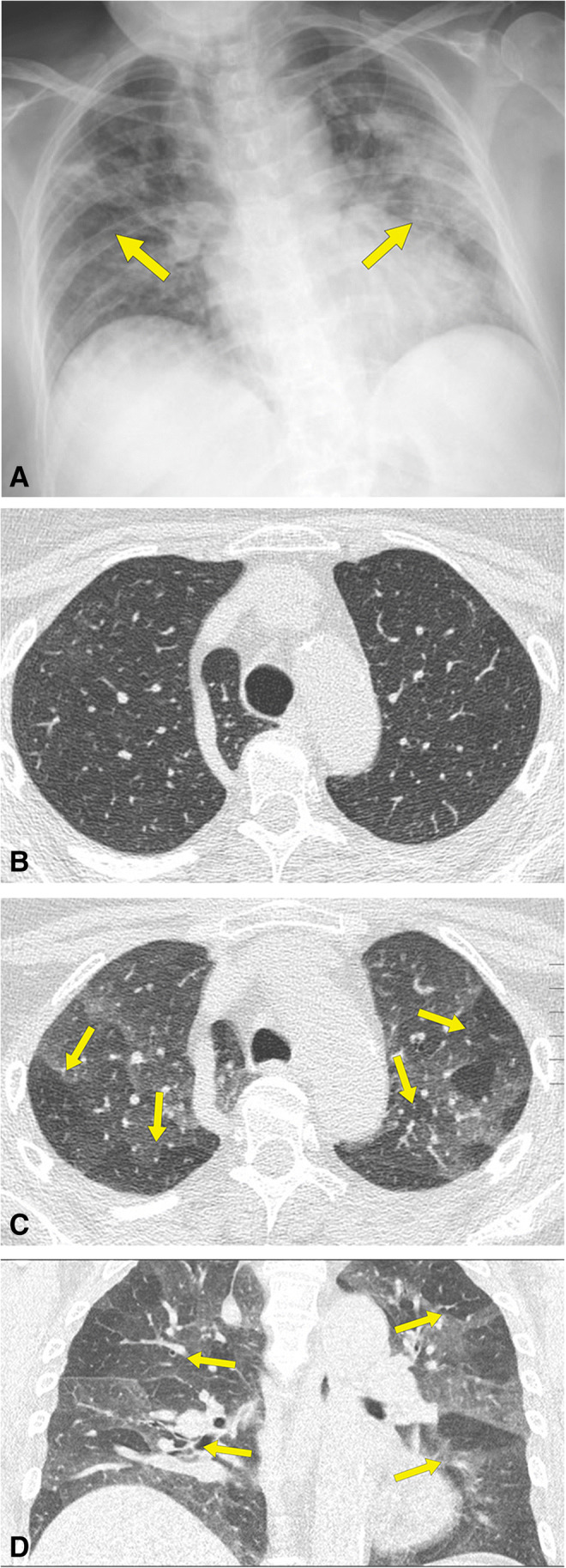
A 67-year-old nonsmoking man who had COVID-19. **A** Anteroposterior chest radiograph shows bilateral and multifocal areas of consolidation (arrows). **B** Inspiratory CT scan at follow-up examination, 65 days after dyspnea and shortness of breath shows no abnormalities. **C** Expiratory CT scan, obtained at same level as **B**, and **D** coronal multiplanar reformation image shows significant bilateral patchy areas of low attenuation due to air trapping (arrows)

Follow-up paired inspiratory/expiratory CT findings in the 48 patients are listed in Table 3. Parenchymal abnormality was found in 50% (24/48) of patients and included air trapping (37/48, 77%), ground-glass opacities (19/48, 40%), reticulation (18/48, 38%), parenchymal bands (15/48, 31%), traction bronchiectasis (9/48, 19%), mosaic attenuation pattern (9/48, 19%), bronchial wall thickening (6/48, 13%), and consolidation (2/48, 4%). The absence of air trapping was observed in 11/48 (23%), mild air trapping in 20/48 (42%), moderate in 13/48 (27%), and severe in 4/48 (8%) (Figs. 1, 2 and 3). The median air trapping score was 11.0 (IQR: 10.0–13.0). Pleural thickening, nodules, round cystic changes, bronchiectasis, pleural effusion, and lymphadenopathy were not observed. Air trapping was associated with ground-glass opacities (*p* = 0.0223) and reticulation (*p* = 0.0034) but not with traction bronchiectasis (*p*=0.0946), parenchymal bands (*p* = 0.1359), mosaic attenuation pattern (*p*=0.0946), consolidation (*p* = 1.0), and bronchial wall thickening (*p* = 0.3132).

**Table 3:** Comparison of thin-section CT parenchymal abnormalities and air trapping at two serial examinations in patients with proven COVID19 infection. A patient may have more than one imaging finding

Findings*	First CT (acute phase) *n* = 9	Second CT (post-COVID)*n* = 48
Ground-glass opacities	7 (78)	19 (40)
Mosaic attenuation pattern	1 (11)	9 (19)
Consolidation	6 (67)	2 (4)
Traction bronchectasis	6 (67)	9 (19)
Reticulation	0 (0)	18 (38)
Parenchymal bands	3 (33)	15 (31)
Bronchial wall thickening	3 (33)	6 (13)
Air trapping	1^✝^	37 (77)

^*^Data are numbers of patients. Numbers in parentheses are percentages

^✝^Only one patient had expiratory thin-section CT to assess air trapping

### Factors associated with air trapping

Air trapping was gender-associated, total air trapping score in men 12.0 (10.0–140) vs. 10.0 (0.0.0–10.0) in women (*p* = 0.0026). It was unassociated with comorbidities. Acute COVID-19 clinical and laboratory parameters associated with air trapping are summarized in Table 4. The interval between the onset of symptoms and admission (*r* = 0.41, *p* = 0.0040), cough (*p* = 0.0037), and ARDS (*p* = 0.0232) was also associated with the degree of air trapping. The degree of air trapping correlated with age (*r* = 0.62, *p* < 0.0001), admission PaO_2_/FiO_2_ ratio (*r* = 0.39, *p* = 0.0054), peak CRP (*r* = 0.34, *p* = 0.0171), and ferritin (*r* = 0.36, *p* = 0.0109). Charlson comorbidity index, initial oxygen saturation, d-dimer, LDH, AST, ALT, KL-6, IL-6, neutrophil and lymphocyte counts, and neutrophil-to-lymphocyte ratio did not correlate with the degree of air trapping. A multivariable logistic regression analysis was performed taking air trapping score as the dependent variable and age, sex, the interval between symptom onset and admission, cough, PaO_2_/FiO_2_ ratio, peak CRP, LDH, d-dimer, IL-6, ferritin, and neutrophil-to-lymphocyte ratio as independent variables. Variables independently associated with the degree of air trapping were gender (OR = 14.92; 95% CI: 1.99–111.71, *p* = 0.0085), and age (OR = 1.14; 95% CI: 1.02–1.26, *p* = 0.0182). Recently, immune-endocrine processes have been proposed for a better understanding in the variations of SARS-CoV-2 pathogenicity. In fact, females mount higher innate immune responses than males, resulting in a faster viral recognition and production of interferon and inflammatory cytokines, inducing more rapid viral clearance [16].

**Table 4:** Demographic, clinical, and laboratory data in patients with and without air trapping

Characteristic	With air trapping (*N* = 37)	Without air trapping (*N* = 11)	*p* value
Age, year	65.0 (56.0 – 74.0)	47.0 (38.0 – 56.2)	0.0005
Male, *n* (%)	31 (83.8)	6 (54.5)	< 0.0001
Charlson comorbidity index	0.0 (0.0 – 1.0)	0.0 (0.0 – 0.0)	0.0328
Comorbidities, %	29 (78,4)	2 (18,2)	0.0005
Arterial hypertension, *n* (%)	20 (54.0)	0 (0.0)	0.0012
Diabetes mellitus, *n* (%)	4 (10.8)	0 (0.0)	0.5607
Other*, *n* (%)	3 (8.1)	2 (18.2)	
Interval onset-admission, days	6.0 (4.0 – 7.0)	7.0 (6.0 – 9.5)	0.0500
Symptoms during acute COVID-19			
Fever, *n* (%)	37 (100)	11 (100)	–
Dyspnea, *n* (%)	22 (59.4)	7 (63.6)	0.9184
Anosmia/ageusia, *n* (%)	10 (27.0)	2 (18.2)	0.7054
Cough, *n* (%)	19 (51.3)	10 (91.8)	0.0324
Fatigue, *n* (%)	7 (18.9)	4 (36.3)	0.2457
Diarrhea, *n* (%)	11 (29.7)	1 (9.1)	0.2475
Arthromyalgia, *n* (%)	2 (5.4)	4 (36.4)	0.0193
Headache, *n* (%)	7 (18.9)	3 (27.3)	0.6753
Rash, *n* (%)	0 (0)	2 (18.2)	–
Abdominal pain, *n* (%)	2 (5.4)	1 (9.1)	0.5507
ARDS, *n* (%)	13 (35.1)	1 (9.1)	0.1388
O_2_ saturation, %	93.0 (92.0 – 96.0)	93.0 (92.0 – 97.0	0.9309
PaO_2_/FiO_2_ ratio	261.0 (176 – 317)	328.0 (285.0 – 414.0)	0.0305
C-reactive protein, mg/L	167.0 (124.3 – 229.0)	81.4(63.1 – 96.3)	0.0024
AST, IU/l	53.0 (31.0 – 118.0)	47.0 (19.7 – 55.0)	0.1046
ALT, IU/l	73.0 (31.0 – 126.0)	61.0 (32.0 – 65.0)	0.4385
LDH, IU/l	367.0 (282.0 – 471.7)	233.0 (148.7 – 504.0)	0.1073
D dimer, ng/mL	638.0 (310.0 – 1080.0)	560.0 (243.0 – 635.2)	0.0749
KL6, U/L	329.5 (141.0 – 548.0)	150.0 (143.0 – 184.0)	0.1461
IL-6, pg/mL	1041.0 (116.0 – 1966.0)	39.1 (19.0 – 112.0)	0.0673
Ferritin, ng/mL	1343.0 (686.0 – 3373.0)	476.0 (104.0 – 769.5)	0.0016
Neutrophil/lymphocyte ratio	5.03 (2.99 – 7.61)	2.21 (1.86 – 5.05)	0.0179
Therapeutics			
Dexamethasone, *n* (%)	31 (83.8)	9 (81.8)	0.7587
Tocilizumab, *n* (%)	15 (40.5)	5 (45,4)	0.9537
Other**, *n* (%)	27 (72.5)	5 (45.4)	0.1440

All values expressed as median and interquartile range (IQR) unless otherwise specified. ^*^Includes dyslipidemia [3], epilepsia [1], and gout [1]

^**^Includes antibiotics [29], hemodialysis [2], and ECMO [2]

*ARDS* adult respiratory distress syndrome, *IQR* interquartile range, *O*_*2*_ oxygen, *PaO*_*2*_ partial pressure of oxygen in arterial blood, *FiO*_*2*_ fraction of inspired oxygen, *AST* aspartate aminotransferase, *ALT* alanine aminotransferase, *LDH* lactate dehydrogenase, *KL6* Krebs von den Lungen glycoprotein, *IL-6* interleukin -6

## Discussion

Our study shows a high prevalence of air trapping in post-COVID patients with respiratory symptoms. Besides, it suggests small-airway disease as its cause and highlights the usefulness of paired inspiration/expiration CT when this condition is entertained.

Among patients who recovered from COVID-19 prolonged symptoms, particularly fatigue and dyspnea are common, and it may take weeks or months for their resolution and return to usual health [2–5]. Recognizing imaging patterns in the follow-up of long-COVID patients is paramount for understanding the pathophysiologic features and natural history of this infection.

Long-COVID symptoms could be related to several results of lung damage including [1] micro-angiopathy, [2] fibrosis, [3] obstructive ventilatory defects, and [4] chronic local and systemic inflammation. Recent COVID-19 follow-up studies have evaluated the presence of microangiopathy and fibrosis [17, 18]. The findings of air trapping in our study suggest that obstructive ventilatory defects and small airway inflammation may play a role in long-COVID pathophysiology.

COVID-19 is characterized by an exuberant inflammatory response caused by overwhelming cytokine release that may predispose patients to thrombotic disease because of excessive inflammation, platelet activation, endothelial dysfunction, and stasis. Among patients with COVID-19, Barnes et al identified the abundant neutrophils and neutrophil extracellular traps (NETs) in the airway and alveolar air spaces [19]. The most noteworthy finding from this study was the discovery of active NETosis in the infected lungs with the persistent release of NETs, observed in bronchiolar and alveolar regions from damaged areas of the lungs even after the virus had cleared [19]. NETs formed in the lungs infected with respiratory viruses represent potential COVID-19 inflammatory lung damage drivers, including small airways, interstitial lesions, and thrombosis leading to fibrin deposition. Air trapping might be attributed either to the capillary wall damage and thickening of small airways walls caused by pro-inflammatory factors [12, 20].

Evaluation of patients following novel coronaviruses, SARS [21], and recently in COVID survivors, have not shown physiologically limiting airflow obstruction. Instead, lung function test (LFT) abnormalities were mainly related to the pulmonary diffusing capacity for carbon monoxide [22, 23]. This is not surprising considering our previous work showing that the extent of air trapping did not correlate with LFT abnormalities, possibly representing bronchiolar inflammation preceding detectable LFT abnormalities [24]. Regional abnormalities in lung ventilation could, however, have a profound effect on ventilation perfusion mismatch in the post COVID lung, particularly given the accumulating evidence for widespread pulmonary vasculopathy in COVID-19 infections [17]. Increased perfusion of underventilated lung may play a major role in driving COVID-19-related hypoxia [25]. Notwithstanding we cannot thoroughly rule out previous bronchiolar damage, the fact that the patients had not any pulmonary disease prior to COVID-19 suggest that the bronchiolar involvement was of inflammatory origin.

In general, underventilation manifesting as air trapping is an important early indicator of small-airway disease (SAD) [26] but limited histological data are available regarding clinical long-COVID-19-related bronchiolitis [27–29]. The association of air trapping with inflammatory biomarkers suggests that SAD may be a manifestation of an inflammatory state. For instance, ferritin, an important biomarker of macrophage activation, positively correlates with air trapping, inflammation, and lung immune-mediated injury in COVID-19 [30].

We have observed air trapping using expiratory CT scans in 77% of our long-COVID patients. Although air trapping on expiratory CT has been observed in long-COVID patients [31–34], it has not been systematically studied in the setting of long COVID with persistent respiratory complaints. Recently, a multicenter study of 108 patients with COVID-19 evaluated the small-airway disease using inspiratory and expiratory chest high-resolution computed tomography. The study detected air trapping following COVID-19 infection but did not show that its presence was associated with the interval since infection [32].

Considering the above, post-infectious SAD is a consideration among patients with SARS-CoV-2 infection presenting with hypoxemia and mosaic perfusion. Although it is not possible to exclude the possibility that some air trapping is attributable to chronic pulmonary embolism [35], we believe that the presence of significant air trapping in these patients probably reflects the presence of persistent bronchiolar inflammation or may correspond to a constrictive bronchiolitis as a sequelae.

In our group of patients, we did not observe enlargement of pulmonary trunk or right-side cardiac cavities.

Although it is not always feasible due to the unstable clinical conditions, especially in critically ill patients, the most widely used imaging technique for assessing air trapping is paired inspiratory and expiratory CT scans. A low-dose CT protocol with iterative reconstruction can be potentially recommended for the follow-up evaluation of symptomatic long-COVID patients.

Our study had several limitations. Firstly, the present study was a retrospective study with small sample size, and future prospective studies with a larger number of patients would be helpful to confirm our results. Secondly, we did not use spirometrically gated inspiratory and expiratory CT, and air trapping was evaluated by using subjective analysis.

Future automated quantification CT will be extremely useful in evaluating an increasing population of post-COVID-19 lung parenchymal changes such as fibrosis or air trapping [36].

In summary, our study suggest that air trapping is a common finding in long-COVID patients with respiratory symptoms. The presence of air trapping on dynamic expiratory CT scans is highly suggestive of obstructive SAD and could reflect the presence of persistent bronchiolar inflammation.

## Abbreviations

AST: Aspartate aminotransferase
COVID-19: Coronavirus disease-19
CPR: C-reactive protein
DLCO: Diffusing capacity for carbon monoxide
ICU: Intensive care unit
IL-6: Interleukin-6
IQR: Interquartile range
KL-6: Krebs von den Lungen glycoprotein-6
LDCT: Low-dose CT
LDH: Lactate dehydrogenase
LFTs: Lung function tests
NETs: Neutrophil extracellular traps
RT-PCR: Reverse transcription polymerase chain reaction
SAD: Small airway disease
SARS-CoV-2: Severe acute respiratory syndrome-coronavirus-2

## Declarations

### Guarantor

The scientific guarantor of this publication is Tomás Franquet MD, PhD.

### Statistics and biometry

One of the authors have significant statistical expertise.

### Methodology

• Prospective

• Observational

• Performed at one institution
